# Supplementary material for: Association between oral microbiome and seven types of cancers in East Asian population: a two-sample Mendelian randomization analysis
Source: Front Mol Biosci. 2023 Nov 21;10:1327893. doi: 10.3389/fmolb.2023.1327893 (PMC10702768; doi:10.3389/fmolb.2023.1327893)
Supplement: Supplementary file 1 [file DataSheet1.ZIP › Supplementary Materials/MR plots of saliva/saliva═╝/Prostate cancer/pheno.2984_to_prostate cancer_forest.pdf]

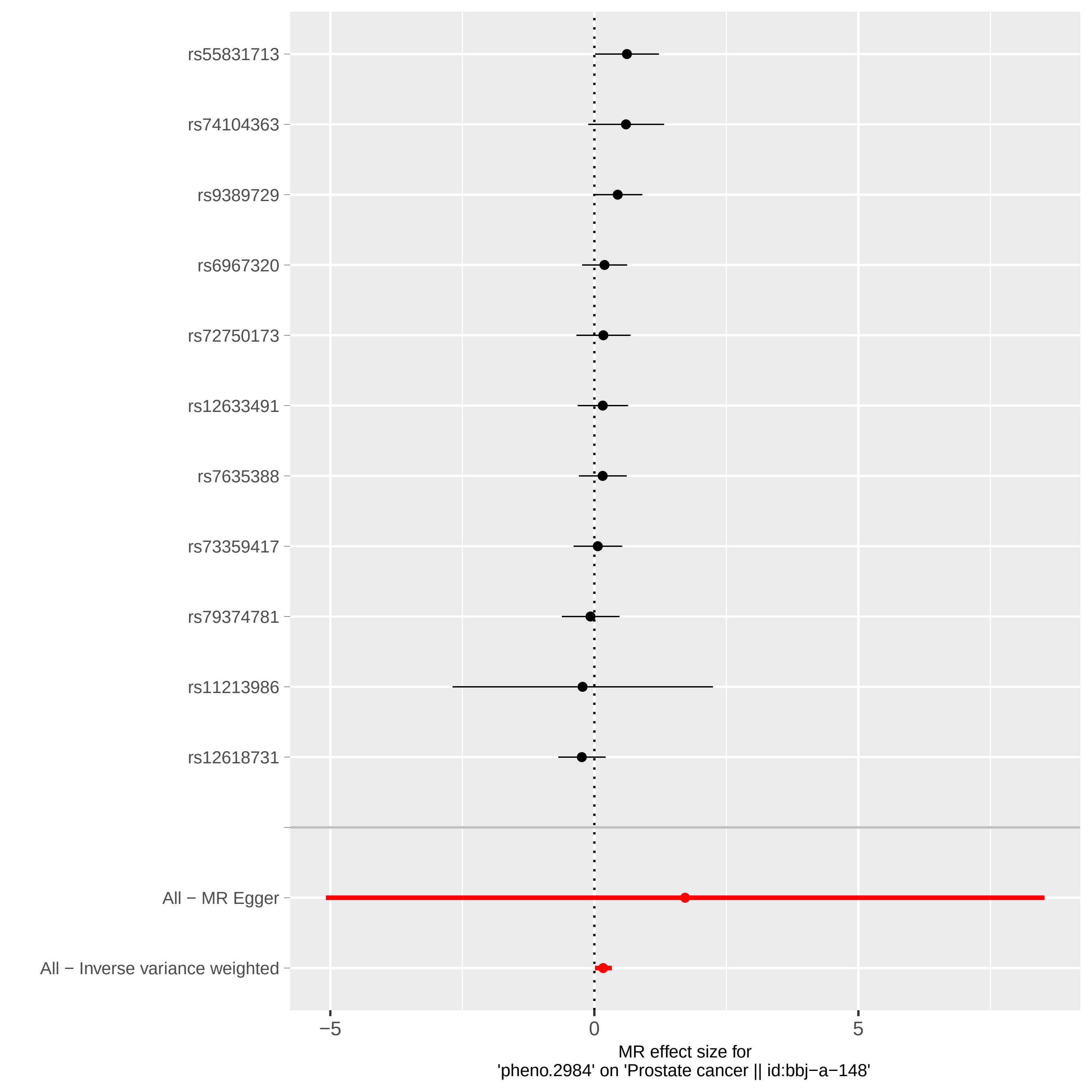
rs55831713
rs74104363
rs9389729
rs6967320
rs72750173
rs12633491
rs7635388
rs73359417
rs79374781
rs11213986
rs12618731
All − MR Egger
All − Inverse variance weighted
−5
0
5
MR effect size for
'pheno.2984' on 'Prostate cancer || id:bbj−a−148'
